# A general strategy for direct, enzyme-catalyzed conjugation of functional compounds to DNA

**DOI:** 10.1093/nar/gky184

**Published:** 2018-03-13

**Authors:** Jochem Deen, Su Wang, Sven Van Snick, Volker Leen, Kris Janssen, Johan Hofkens, Robert K Neely

**Affiliations:** 1 Laboratory of Molecular Imaging and Photonics, Department of Chemistry, KU Leuven, Celestijnenlaan 200F, 3001 Heverlee, Belgium; 2 School of Chemistry, University of Birmingham, Edgbaston, Birmingham B15 2TT, UK

## Abstract

The methyltransferase enzymes can be applied to deliver a range of modifications to pre-determined sites on large DNA molecules with exceptional specificity and efficiency. To date, however, a limited number of modifications have been delivered in this way because of the complex chemical synthesis that is needed to produce a cofactor analogue carrying a specific function, such as a fluorophore. Here, we describe a method for the direct transfer of a series of functional compounds (seven fluorescent dyes, biotin and polyethylene glycol) to the DNA duplex. Our approach uses a functional cofactor analogue, whose final preparative step is performed alongiside the DNA modification reaction in a single pot, with no purification needed. We show that fluorophore conjugation efficiency in these mixtures is significantly improved compared to two-step labeling approaches. Our experiments highlight the remarkable malleability and selectivity of the methyltransferases tested. Additional analysis using high resolution localization of the fluorophore distribution indicates that target sites for the methyltransferase are predominantly labeled on a single strand of their palindromic site and that a small and randomly-distributed probability of off-site labeling exists.

## INTRODUCTION

DNA methyltransferase enzymes (MTases) catalyze the transfer of methyl groups from the ubiquitous cofactor, *S*-adenosyl-l-methionine (AdoMet), to DNA. There are many thousands of known and putatitive DNA methyltransferases whose targets lie within short DNA motifs typically four to eight base pairs in length. Within these motifs, the methyltransferases target either cytosine (at C5 or N4) or adenine (at N6) for methylation (1).

**Figure 1.**
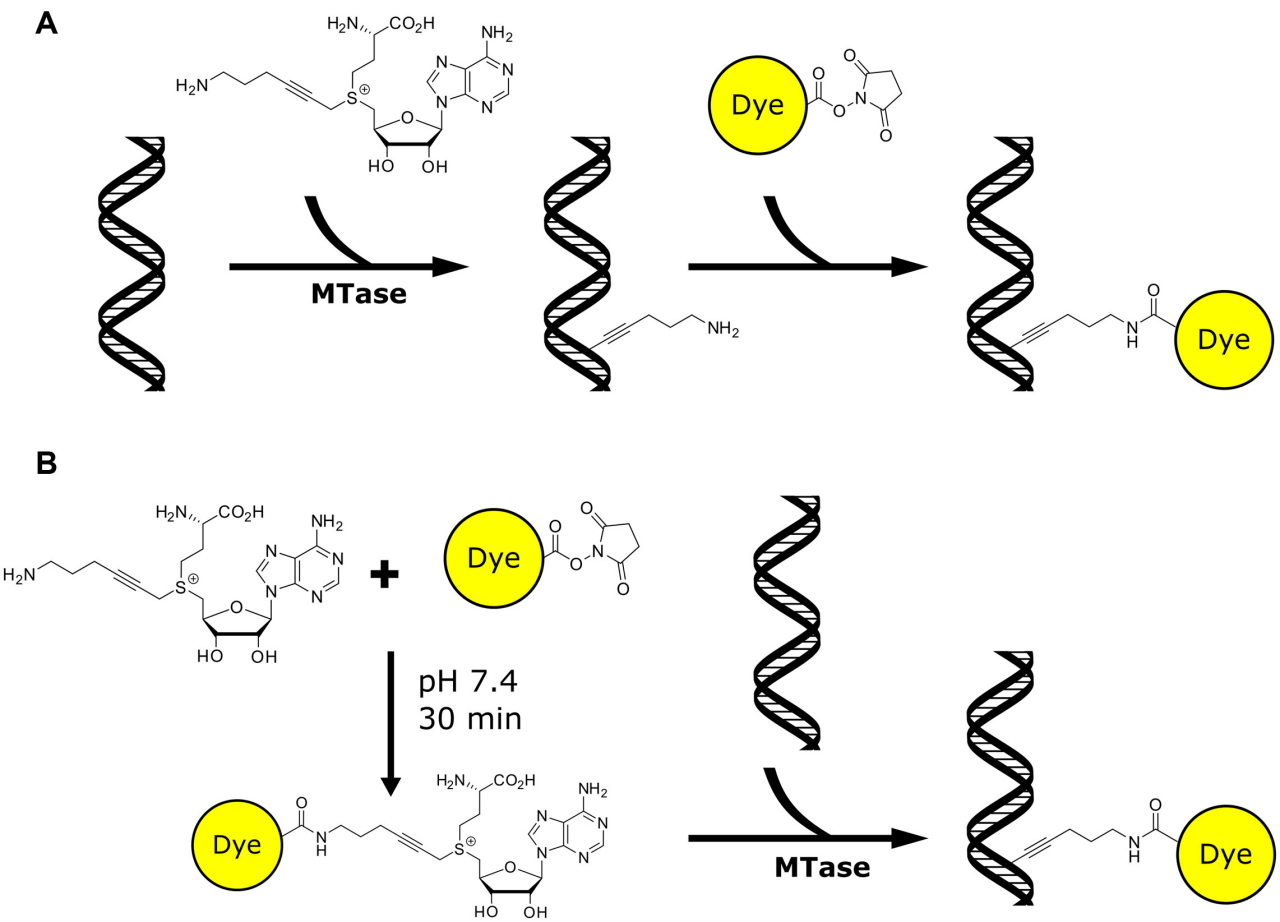
General labeling scheme for addition of fluorophores to the DNA. (**A**) 2-Step, mTAG conjugation of fluorophores to DNA. In the first step a reactive moiety is transferred to the DNA by DNA MTases, in a subsequent reaction the dye is coupled to the DNA. (**B**) One-pot labeling of DNA. An NHS-ester derivative (e.g. fluorophore) is coupled directly to the AdoMet analogue, this mixture is then incubated with methyltransferase and DNA. Hence, a fluorescent moiety is directly transferred to the DNA by the methyltransferase enzyme.

The MTases can also catalyse efficient transalkylation of DNA, where their AdoMet cofactor is replaced by a synthetically-prepared AdoMet analogue that carries a substantially more complex transferable moiety than a methyl-group (2–4). This method has been termed methyltransferase-directed Transfer of Activated Groups (mTAG). The standard mTAG reaction enables fluorescent labeling of DNA by a two-step procedure, as depicted in Figure 1A. The first step is the DNA MTase catalyzed transalkylation reaction- the transfer of a chemically-reactive moiety, e.g. alkyne (5–7), azide (8,9) or amine (2,6,10), from AdoMet analogue onto DNA. This is followed by a more standard coupling reaction to ‘functionalize’ the DNA (e.g. using an NHS-ester-conjugated fluorophore) (3,7,10,11).

This approach for DNA modification has found application in sorting of methylated and un-methylated DNA for subsequent analysis by sequencing (9) and in DNA mapping experiments (10,12–14). In both cases, the quality of the information derived from these experiments depends on the underlying DNA modification efficiency and specificity. Whilst enzymatic efficiency of methyltransferase is typically high (15,16), the subsequent conjugation of a fluorophore (or other functional moiety, such as biotin) to the DNA can be rather less efficient.

This problem can be circumvented by using the methyltransferase to directly transfer a functional group to the DNA. Indeed, such an approach has been reported using a reactive aziridine-based cofactor analogues (17) and by Grunwald *et al*. (12), who synthesized an AdoMet analogue carrying a tetramethylrhodamine (TAMRA) moiety that was directly transferred to DNA using a methyltransferase-catalysed reaction. Additionally, more recent results demonstrated the transfer of photo-caged groups using DNA methyltransferases (18). These approaches carry significant risk (and cost), however, since the final step of the synthesis of the AdoMet analogue is a rather inefficient reaction for coupling the transferable moiety to *S*-adenosyl-l-homocysteine. To compound matters, current knowledge of the design principles for functional AdoMet analogue/methyltransferase partners is limited. Hence, in order to build a range of functional cofactor analogues that can be combined with a range of methyltransferase enzymes for efficient, single-step DNA modification a more effective means of producing and screening cofactor analogues is needed.

We sought to achieve this by producing a range of AdoMet analogues in a one-pot reaction with the DNA methyltransferases and their DNA targets. We develop this approach with a series of fluorescent molecules and compare our one-pot reaction with the alternative two-step labeling methodology. We derive optimized reaction conditions for the chemistries we investigated, and show that the one-pot labeling approach is an extremely efficient, bio-orthogonal route to couple a multitude of functional groups directly to DNA.

## MATERIALS AND METHODS

All chemical reagents were purshased from Sigma Aldrich and used according to the manufacturer's instructions, unless stated otherwise in the text. The DNA methyltransferase enzymes (M.TaqI, M.FokI, the triple mutant (Q82A/Y254S/N304A) of M.HhaI and double mutant (Q136A/N374A) of M.MpeI) have been prepared as described previously (7). All synthetic AdoMet analogues were prepared according to the method reported by Lukinavičius *et al*. (8,9). Reagents for performing methyltransferase-directed DNA labeling are available from Chrometra (Leuven, Belgium).

### Preparation of sequence-specifically functionalized plasmid DNA for amine-to-NHS-ester coupling

The reaction mixture for a final volume of 100 μl contained 10 μg of pUC19 plasmid, 10 μl of 10× CutSmartTM buffer (NEB), 120 μM Ado-6-amine cofactor (9,13), 2 μl of the enzyme M.TaqI (1.412 mg/ml), and Milli-Q water (Synergy UV, Merck-Millipore) to complete the volume. This system was incubated for 2 h at 60°C. After which, 1 μl of proteinase K was added and the reaction mixture was incubated for 1 h at 55°C. The preparation was purified using silica-based columns (DNA Clean & Concentrator – 5 kit, ZymoResearch) and eluted in 25 μl of Milli-Q water.

### Site-specific mutagenesis

The M.TaqI recognition sequence 5′-TCGA-3′ was mutated into 5′-TCGG-3′. The multiple cloning site that contains two M.TaqI recognition sites were cut out completely with restriction enzyme EcoRI (Fermentas) and PstI (Fermentas) in 1× FastDigest buffer (ThermoFisher), which was then subjected to gel electrophoresis (with 1% agarose gel). The correct band (2629 bp) was cut out of the gel and purified with Gel Extraction Kit (Fermentas). An oligo with the sequence of 5′-GA GGA GGA ATT CGG GCT CGG TAC CCG GGG ATC CTC TAG AGTC GGC CTG CAG GAG GAG-3′ (which was identical to the multiple cloning sites but contained no M.TaqI recognition sequence)) was ordered from IDT, cut with EcoRI (Fermentas) and PstI (Fermentas) restriction enzymes, and purified with PCR purification kit (Fermentas). These two fragments were then ligated with T7 ligase (Thermo Scientific), and transformed into T7 competent cells (NEB). The selected colonies were cultured and the mutated plasmids were extracted by Miniprep kit (Ferments). The final sequence was confirmed by sequencing (LGC genomics).

The other two M.TaqI recognition sites were mutated one after one via site specific mutagenesis procedure (Q5 Sit-Directe Mutagenesis Kit, NEB). Used primers were: 5′-GCA CGA GTG GGT TAC ACC GAA CTG GAT CTC AAC-3′; 5′-GTT GAG ATC CAG TTC GGT GTA ACC CAC TCG TGC-3′; 5′-GAG CA TCA CAA AAA TCG GCG CTC AAG TCA GAG GTG-3′ and 5′-CA CCT CTG ACT TGA GCG CCG ATT TTT GTG ATG CTC-3′. The success mutations were confirmed by sequencing (LGC genomics).

### PCR-reaction

The 5 kb fragment was amplified from the T7 bacteriophage genome, using the Q5 High fidelity 2× master mix (NEB). Both the forward primer (5′-CG AGT CCT CCA AGA TGG-3′) and reverse primer (‘5-CC TCT CCC TAT AGT GAG TCG-3′) were ordered from Integrated DNA Technologies. In the final PCR reaction mixture 500 nM of the primers were incubated with 0.5 ng of T7 DNA with the following scheme: Denaturation at 98°C for 30 s, Annealing at 64°C for 15 s and extension at 72°C for 2 min 20 s for 25 cycles.

### Fluorescent labeling using the amine-to-NHS-ester coupling

A 50 μl reaction contains 0.89 μg amine modified pUC19, 0.01 M PBS buffer, 500 μM Atto-647N-NHS dye (Atto-Tec) and the volumes of DMSO corresponding to 5, 10, 25 and 30%. The reactions were incubated for 2 h at room temperature and purified by silica-based columns (DNA Clean & Concentrator – 5 kit, ZymoResearch). The fluorescently labeled DNA was eluted using the elution buffer provided by the manufacturer.

### Direct DNA modification using the fluorophore-coupled cofactor

Initially, the Ado-6-amine cofactor was coupled to the NHS-ester dye. Therefore, in a 5 μl reaction, 0.01 M PBS buffer, 0.8 mM Ado-6-amine cofactor (13,19) and 2.4 mM Atto-647N-*N*-hydroxysuccinimidyl (NHS)-ester were mixed and kept on ice for 30 min. Then, 2 μl of NEB 10× Cutsmart™ buffer was added to quench the reaction. After 1 minute on ice, 2 μl of 1 μg/μl pUC19 plasmid or hairpin DNA, 0.5 μl of M.TaqI enzyme (1.412 mg/ml) and 11.5 μl of Milli-Q water were added. The system was incubated at 60°C for 1 h. 1 μl of proteinase K was added to the reaction and was followed by incubation at 55°C for 1 h. The purification of pUC 19 plasmid was performed with a silica-based column (DNA Clean & Concentrator – 5 kit, ZymoResearch), while the hairpin DNA was purified with a column that was charged with Sephadex G-50 in STE buffer (10 mM Tris–HCl, pH 7.5, 1 mM EDTA, 100 mM NaCl, mini Quick Spin DNA Columns, Sigma-Aldrich).

### Restriction digestions

1 μg of DNA was labeled according to the protocol above. After purification, the DNA was incubated with 1μl of R.Taq^α^I (NEB) for 1 h at 60°C. Subsequently, the mixture was incubated with 1 μl of proteinase K (NEB) for 1 h at 55°C and purified again using the silica-based column (DNA Clean & Concentrator – 5 kit, ZymoResearch). After this, the mixture was placed in a 1% agarose gel and run at 120 V for 25 min. For the control samples with AdoMet (NEB), we used the supplier's recommended amount (80 μM).

### Mass spectrometry

The labeled hairpin samples were subjected to MALDI, LC–MS (Waters Maldi Synapt G2 TOF Mass Spectrometer). The applied method was time-of-flight (TOF) mass spectrometry with electrospray ionization. 1% TAE in water/acetonitrile was used as running buffer.

### DNA deposition

For the microscopic measurements on plasmid DNA, the DNA was spincoated onto poly-l-lysine (PLL) coverslips. Firstly, the coverslips were baked in 450°C oven for 48 h. Secondly, 70–100 μl of 0.01% (v/v) poly-l-lysine in water was dropped on a coverslip and allowed to incubate at room temperature for 15 min. Subsequently, the coverslips were washed thoroughly with Milli-Q water and dried under argon flow. Fourthly, 50 μl DNA sample containing 25 ng DNA, 10 mM Tris (pH 7.4) was spincoated onto the PLL modified coverslips at 3000 RPM for 90 s. The DNA sample was added once the spinning started. Finally, immediately after the previous step, the sample was washed with 5 ml of Milli-Q water at 5000 RPM for another 90 s.

For the high resolution measurements, linear DNA was stretched on zeonex coverslips following the method from Deen *et al*. (20). The coverslips were spincoated with a solution of 1.5 wt% Zeonex (Zeonor) in toluene. Subsequently, the coverslips were dried in the oven at 110°C for 2 h. The DNA was diluted to a concentration of 0.2–1 ng/μl in 50 mM MES (Sigma-Aldrich) solutions which contained 100 nM YOYO-1 (invitrogen). The solution was incubated for 10 min at 50°C before stretching by a rolling droplet, with a speed of 2 mm/min.

### Imaging

A perfusion chamber (Grace Bio-labs) was placed on top of the spin-coated plasmid DNA, and 50 μl of imaging buffer consisting of 5 nM YOYO-1 and 50 mM MEA (Sigma-Aldrich) was added to the sample. All samples were imaged using an olympus IX-83 wide field Microscope with a 150× TIRFM objective and an NA of 1.45 (Olympus). The samples were excited by a 200 mW 488 nm and 100 mW 640 nm diode laser (Olympus) using a quad band (405/488/561/635) dichroic filter. Emission was collected via a quad band (25 nm band pass 446/523/600/677) emission filter coupled to a Hamamatsu EM-CCD X2 camera.

Imaging was perfomed as described previously (20,21). Briefly, for the collection of the plasmid images, the sample was first imaged with 640 nm laser light to detect the Atto647N dye for approximately 1000 frames (exposure time 30 ms). Subsequently, using TIRF microscopy, the YOYO-1 was bleached for 15 s using 488 nm laser light, followed by imaging for ∼2000 frames with 30 ms exposure time. Approximately 10–15 movies were recorded for randomly selected regions for each sample.

### Image analysis

Localization of dyes on a DNA fragment was done by bleaching localization of fluorophores described earlier by Neely *et al.* (10). Briefly, the method is based on the stochastic nature of photobleaching of single dyes. In the last frame of the movie only single isolated fluorophores exist that can be fitted by a 2D Gaussian profile. By subtracting this Gaussian profile from all previous frames in the movie, closely aligned sites can be resolved.

### Counting labels on plasmids

Fluorophore counting and super-resolution imaging analysis was carried out using Localizer software (19). The imaging experiment was performed in a manner similar to that we recently reported (21). In brief, we use reversible binding of YOYO-1 dyes to reconstruct a high resolution image of each DNA plasmid molecule. The image is then converted into a binary image by setting a threshold. We disregard plasmids that are not circular in appearance and count the number of localized, bleached molecules (see above) within the perimeters of the remaining plasmids. Then we created a histogram for each experiment. To determine the average number of labels we simply divided the total number of labels associated with plasmids by the number of plasmids. The standard deviation of the mean (stdev(*X*)/√*N*, where *X* is the histogram data and *N* the number of plasmids) reflects the error of the mean.

### Mapping the PCR fragment

The stretched 5 kb PCR fragments were aligned to the reference sequence with a stretching value of 1.6 using a modified version of the Smith–Waterman algorithm described in (22). The algorithm assigned a positive score to closely matching labels with a width of 150 bp and a stretching variation of 0.1.

Following alignment, all matched labels were aligned to the sites on the reference, with the remaining labels distributed according to the distance from the aligned labels based on localization data. To remove dual stranded labels, for each aligned labels, a single unaligned label within 100 bp was removed from the final distribution.

## RESULTS AND DISCUSSION

We tested a one-pot approach that allows the rapid generation of a range of AdoMet analogues, with different functionalities on the transferable arm of the molecule, and their subsequent application methyltransferase-directed DNA transalkylation, as shown in Figure 1B. We added three equivalents of an *N*-hydroxysuccinimidyl (NHS)-ester fluorophore derivative to the Ado-6-amine analogue, allowing the formation of the cofactor. Following this, we quench the reaction for unreacted NHS-ester groups through the addition of an excess of buffer containing 2-amino-2-(hydroxymethyl)propane-1,3-diol (Tris). This allows us to be confident that any subsequently observed fluorophore conjugation to DNA is due to the direct, methyltransferase catalyzed reaction and not due to the mTAG reaction with Ado-6-amine, followed by an NHS-ester-fluorophore conjugation. Finally, we rely on the supreme specificity of the methyltransferase enzyme to catalyse the transalkylation reaction using the newly formed AdoMet analogue in this mixture. We simply add DNA and the methyltransferase to the reaction mixture and incubate to produce fluorescently labeled DNA molecules.

We tested this method with a number of well known fluorophores (Atto647N, Atto565, TAMRA, Atto520, Atto488, Alexa488), as well as biotin and a large 5000 MW PEG moiety. Initial tests (and work throughout this manuscript, unless otherwise stated) were performed using the prototypical M.TaqI enzyme (TCGA). However, we have been able to functionalize DNA using wild-type M.FokI (GGATG) (Supplementary Figure S6), a triple mutant (Q82A/Y254S/N304A) of M.HhaI (GCGC) (Supplementary Figure S7) and a double mutatant (Q136A/N374A) of M.MpeI (CG) (Supplementary Figure S8).

In all cases, initial analysis of DNA transalkylation efficiency was carried out using a restriction digestion. Here, where transalkylation is complete, DNA restriction (cutting) is blocked. We found complete protection of the DNA for M.TaqI-directed transfer of Alexa 488, Atto 520, Atto 565 and Atto 647N, as well as biotin and the 5000 MW PEG polymer. The transfer of both TAMRA and Atto 488 by M.TaqI led to partial protection (Supplementary Figures S1–S5).

Resistance of modified DNA against restriction digestion, allows us to be confident that DNA modification has occurred at the sites for a given restriction enzyme. However, it does not confirm that fluorescent labeling has been successful. In order to verify this on plasmid DNA, we labeled a 2.6 kb plasmid DNA molecule (pUC19) using the M.TaqI methyltransferase enzyme and Atto647N. Following labeling the plasmid is purified using a PCR clean-up kit and then deposited on a poly-l-lysine-coated glass coverslip and imaged using TIRF microscopy in two colors. The first color is used to describe the shape of the DNA with the intercalating dye YOYO-1, while the second color was used to count the number of labels bound to the DNA by the mTAG reaction, as we described previously (21). Using this approach, we are able to count fluorophores on thousands of individual plasmid molecules and hence, develop a clear picture of labeling efficiency arcoss the ensemble of DNA molecules. The pUC19 plasmid contains four recognition sites for M.TaqI and since these sites are palindromic, each site can potentially carry two fluorophores and hence up to eight fluorophores can be attached to a single plasmid.

### Direct MTase-directed labeling

**Figure 2.**
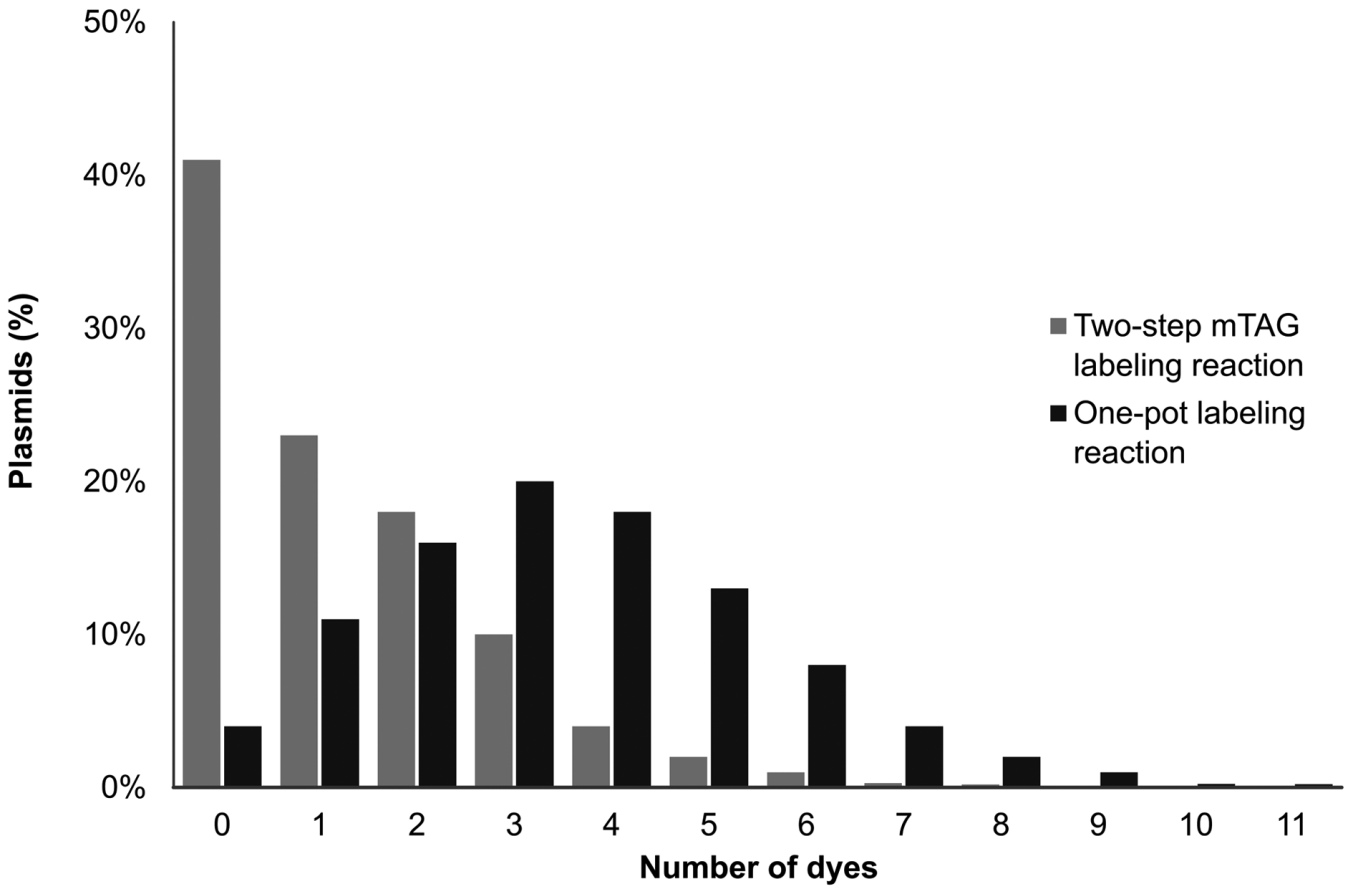
Distribution of localized Atto647N labels on the plasmids for the standard two-step mTAG labeling reaction (light-gray) compared with the one-pot labeling reaction (black). The average number of fluorophores per plasmid in the standard reaction is 1.3 as compared to 3.5 for the one-pot reaction.

We compared the labeling efficiency of the 2-step mTAG reaction to the one-pot approach. As we noted before (21) and is shown in Figure 2, fluorophore (Atto647N) coupling using the 2-step (amine-NHS ester) coupling is rather inefficient. In the best case, ∼40% of the plasmid molecules carry no fluorophore following this reaction. This result is consistent with our earlier DNA mapping experiments, where we found that even the brightest DNA molecules that we selected for mapping carried fluorophores at up to half of their available labeling sites. We submitted DNA, labeled using the one-pot reaction, to our fluorophore counting assay for measuring the labeling efficiency. This revealed a dramatic, 2.6-fold increase in the average number of labels per plasmid for the one-pot reaction compared to the two-step method (Figure 2).

This one-pot reaction shows a labeling efficiency that exceeds any of the two-step chemistries that we have previously reported and demonstrates the remarkable ability of the M.TaqI methyltransferase to direct DNA transalkylation with AdoMet analogues carrying a range of bulky, charged and lipophilic moieties. However, our expectations for the one-pot reaction were that this direct, enzyme catalyzed transfer of a fluorophore would result in DNA labeling with near 100% efficiency, i.e. eight labels per pUC19 plasmid molecule. Hence, we sought to further understand the parameters limiting labeling efficiency by optimizing the conditions for the one-pot labeling reaction.

### Optimization of the one-pot labeling reaction

Initially, we varied the conditions of the dye (Atto647N-NHS ester)-to-cofactor coupling reaction, Supplementary Figure S9. We found no dependence of labeling efficiency on cofactor concentration (above 100μM) and a very minor improvement in coupling efficiencies going from 12.5× > 3× > 1.5× excess of Atto647N in the coupling reaction. Increasing the coupling reaction times from 10 to 20 min (at 4°C) lead to a significant improvement in labeling efficiencies, however increasing it further to 90 min resulted in no drastic improvements (Supplementary Figure S10). Hence, after 20 min the coupling reaction can be assumed to have run to completion.

**Figure 3.**
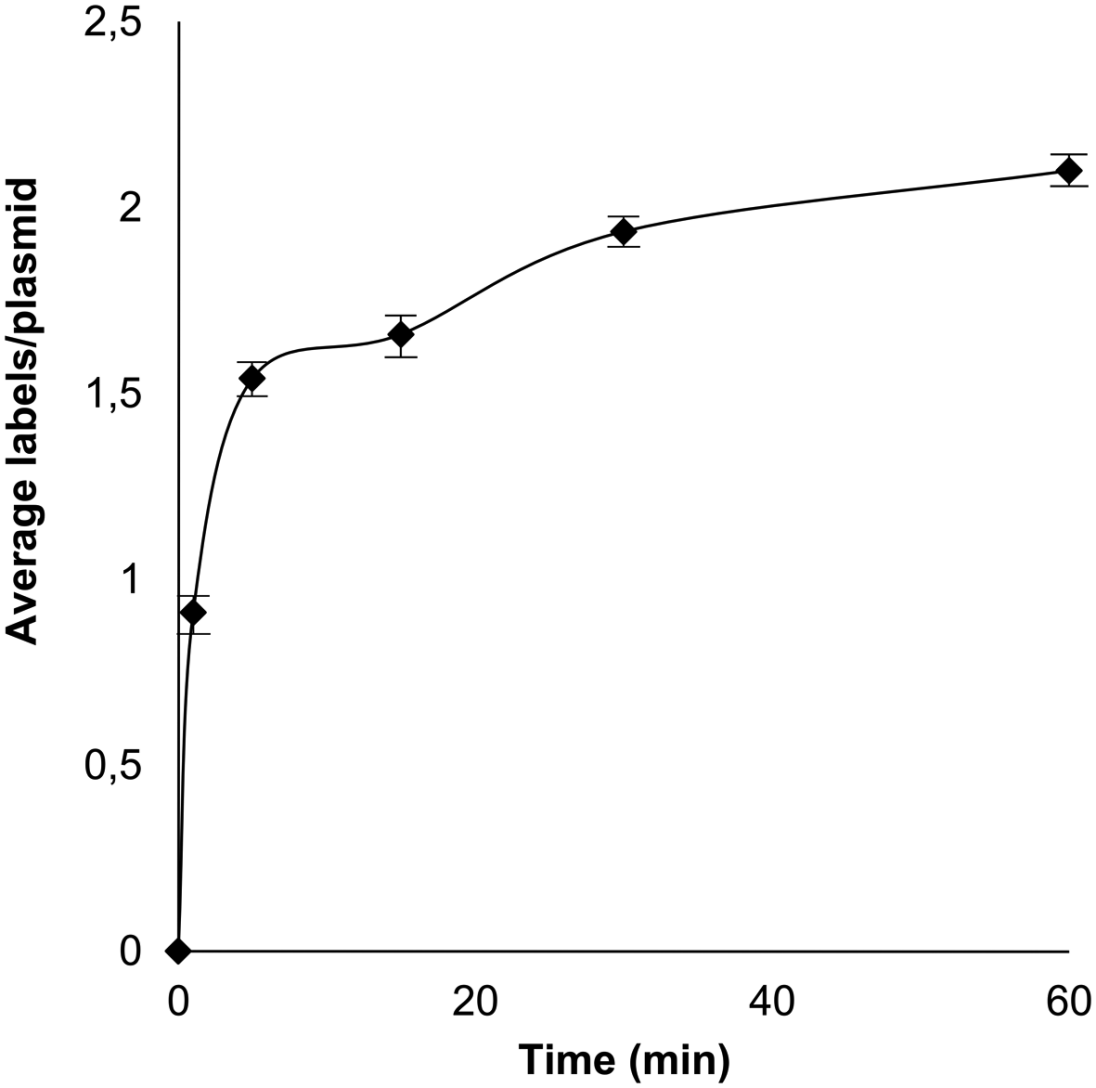
Rate of increase of the average number of localized Atto647N labels per plasmid. The average number of labels per plasmid increased dramatically in the first 10 min of the reaction, after which the reaction slowed until reaching a plateau around 1 h.

We were able to follow the kinetics of the alkylation reaction using the newly-formed, fluorescent AdoMet analogue in a one-pot reaction by stopping the reaction after a set time. We found the methyltransferase is able to efficiently catalyze the transalkylation reaction in this mixture (with a pseudo first-order rate constant of *k*′ = 0.28 min^−1^) and that after 30 min the reaction has neared completion (Figure 3). We also examined the M.TaqI-directed translkylation reaction and the impact of varying it's conditions on DNA labeling efficiency for the one-pot reaction. While the low reaction temperature reaction of 30°C resulted in a slightly less efficient labeling (2.4 labels per plasmid), increasing the reaction temperature further from 40–60°C resulted in only marginal improvements in labeling efficiencies (from 3.2 to 3.7 labels per plasmid, Supplementary Figure S11), presumably as a result of an improvement in the enzymatic reaction rate (1 h reaction). Decreasing the pH of the reaction should lead to improved stability of the Ado-6-amine cofactor and indeed, we see a slight improvement (3.2 labels per plasmid compared to 2.7) in labeling efficiency at pH 7, as compared to pH 7.9 (Supplementary Figure S11).

### Specificity of DNA transalkylation

In a typical population of DNA molecules, we consistently observe a small fraction (1–2%) of the plasmids, which contain more than the expected number of fluorophores (>8). To investigate the reason for this, we performed fluorophore counting experiments with Atto647N-NHS ester, which we incubated plasmid DNA and M.PvuII or M.BsaHI, which are inactive with respect to the Ado-6-amine cofactor. Following DNA purification, in both experiments we found that >97% of the DNA was free of fluorophores. Hence, we conclude that the probability of non-specific dye association with the DNA is less than one fluorophore per ∼85 kb.

**Figure 4.**
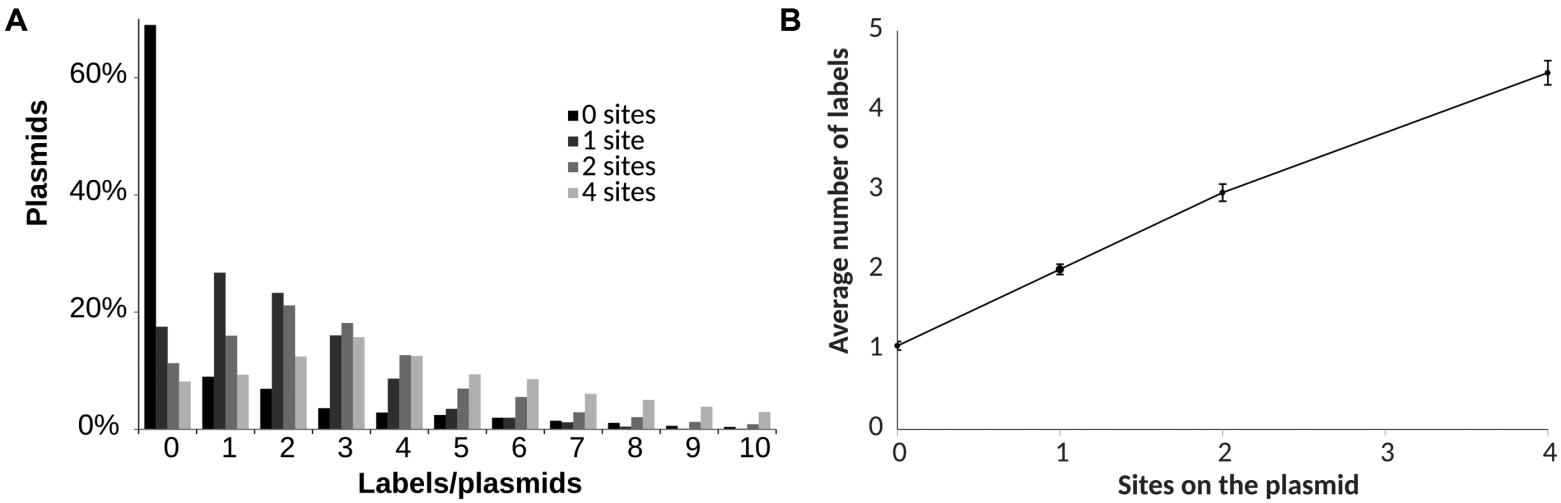
Non-specific labeling of plasmid DNA using the one-pot transalkylation reaction for M.TaqI/Ado-6-amine/Atto647N. (**A**) Distribution histogram of plasmid DNA containing various labels per plasmid. The majority of the plasmid DNA molecules in each group contain the corresponding M.TaqI recognition sites on the plasmid. (**B**) The average number of labels per plasmid compare to the M.TaqI recognition sites on the plasmid. The false positive labeling introduces around 1 extra label per plasmid molecule.

In light of this, we went on to investigate susceptibility of M.TaqI to perform off-target DNA transalkylation reactions. We created mutated pUC19 plasmid DNA molecules with varying numbers of M.TaqI target sites (TCGA) and added these to our one-pot reaction with M.TaqI and the Ado-6-amine/Atto647N conjugate. Following DNA purification, we observe some off-site fluorophore conjugation. Indeed, plotted as the number of counted labels versus number of sites available on a plasmid (Figure 4), we observe a trend that relates the former, to the latter plus one. Hence, on average, we see one off-target label on each DNA plasmid; approximately four off-target labels per 10 kilobase pairs of DNA.

**Figure 5.**
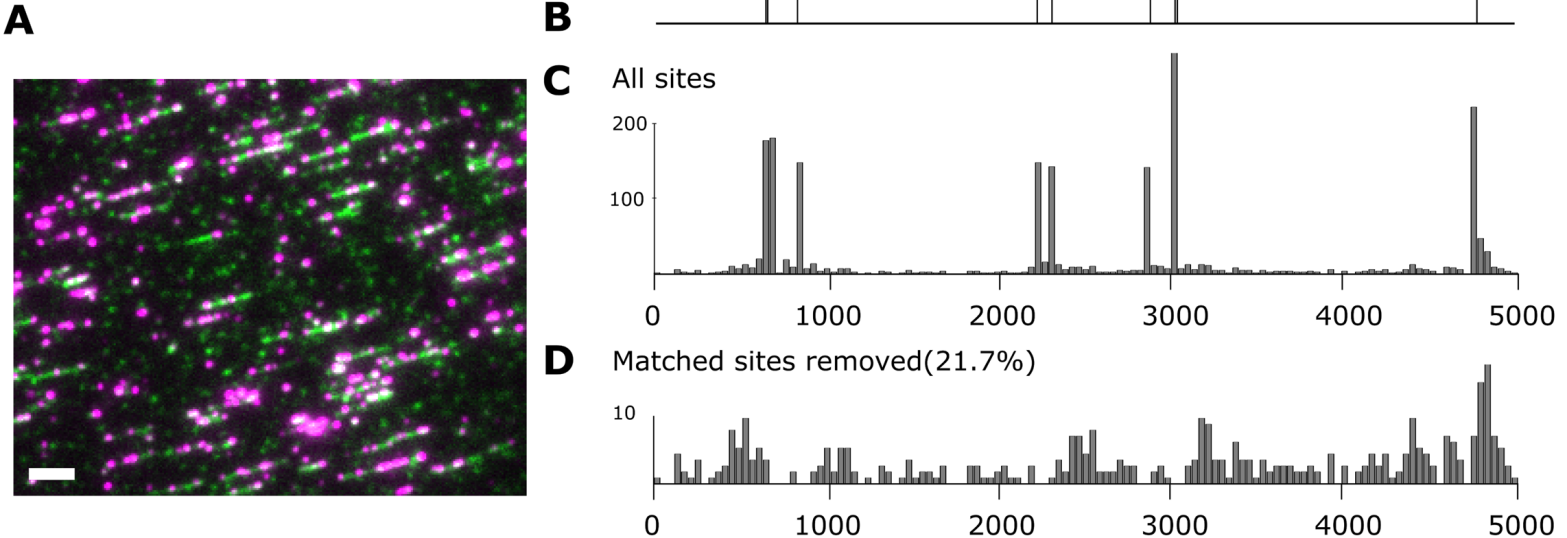
M.TaqI labeling on short PCR fragments. (**A**) Dual colour wide-field microscopy image of the short PCR fragment, the DNA molecules were labeled with M.TaqI and Atto647N (presented in red colour), and in addition, intercalating dye YOYO-1 was used (presented in green colour) to outline the DNA molecules. The white bar is 5 kb. (**B**) Theoretical map with 9 labels on the fragment. (**C**) Consensus map of M.TaqI labeled PCR fragment. The position information of dyes on 180 molecules were aligned and subsequently combined to generate the averaged position map for the labels. (**D**) Distribution of remaining sites when removing the matched sites.

We sought to further investigate the location of off-site fluorescent labeling and to do this we used PCR to create a short 5 kb fragment of DNA and deposited this onto an optically transparent polymer (Zeonex 350R) using molecular combing (20). Following deposition, we recorded super-resolution images of the molecules (as described previously (10)), Figure 5).

The locations of fluorophores along DNA molecules can be determined with a precision of the order of 100 bp using this approach. We extracted such lists of positions for 180 molecules and aligned these to a reference map of the known M.TaqI sites on the short PCR fragment. By combining these individual alignments, we were able to produce a histogram displaying a ‘consensus map’ of the PCR fragment in which the number of counts at a given coordinate along the DNA are plotted in a simple histogram, Figure 5A.

To quantify the extent of- and to investigate any sequence specificity of the off-site labeling by M.TaqI—we automatically removed all the matched sites from the consensus map by removing a single site lying within 100 bp of a known M.TaqI site. This results in the distribution of ‘off-site’ labeling events, as shown in Figure 5D.

The off-site labeling by M.TaqI represents a significant fraction of the total number of fluorophores located on the PCR fragments (21.7%). This equates to ∼0.46 spurious fluorophores per kilobasepair of DNA for this 5 kb fragment, in excellent agreement with the observed off-site labeling for the pUC19 plasmids we examined (0.37 labels/kb). However, these fluorophores are evenly distributed across the entire DNA molecule and as a result contribute significantly <10% of the intensity of those fluorophores located at the target M.TaqI sites to the final histogram.

Furthermore, the M.TaqI target sites of the DNA sequence are proportionately represented in the final consensus map, indicating no observable bias in the labeling approach. For example, the two regions in the map that have two closely aligned sites (at 650 and 3000 bp) both have counts with twice the number of the isolated sites. The excellent matching of the consensus map is particularly encouraging given the fact that very few of the DNA fragments contributed all nine sites to the consensus.

Finally, we sought to address the question of why the data from fluorophore counting on plasmid DNA molecules reveals distributions of fluorophores that are substantially less than the expected eight fluorophores per pUC19 molecule. In particular, we hypothesized that M.TaqI is generally only capable of labeling at one or other of the available bases in its palindromic target site.

**Figure 6.**
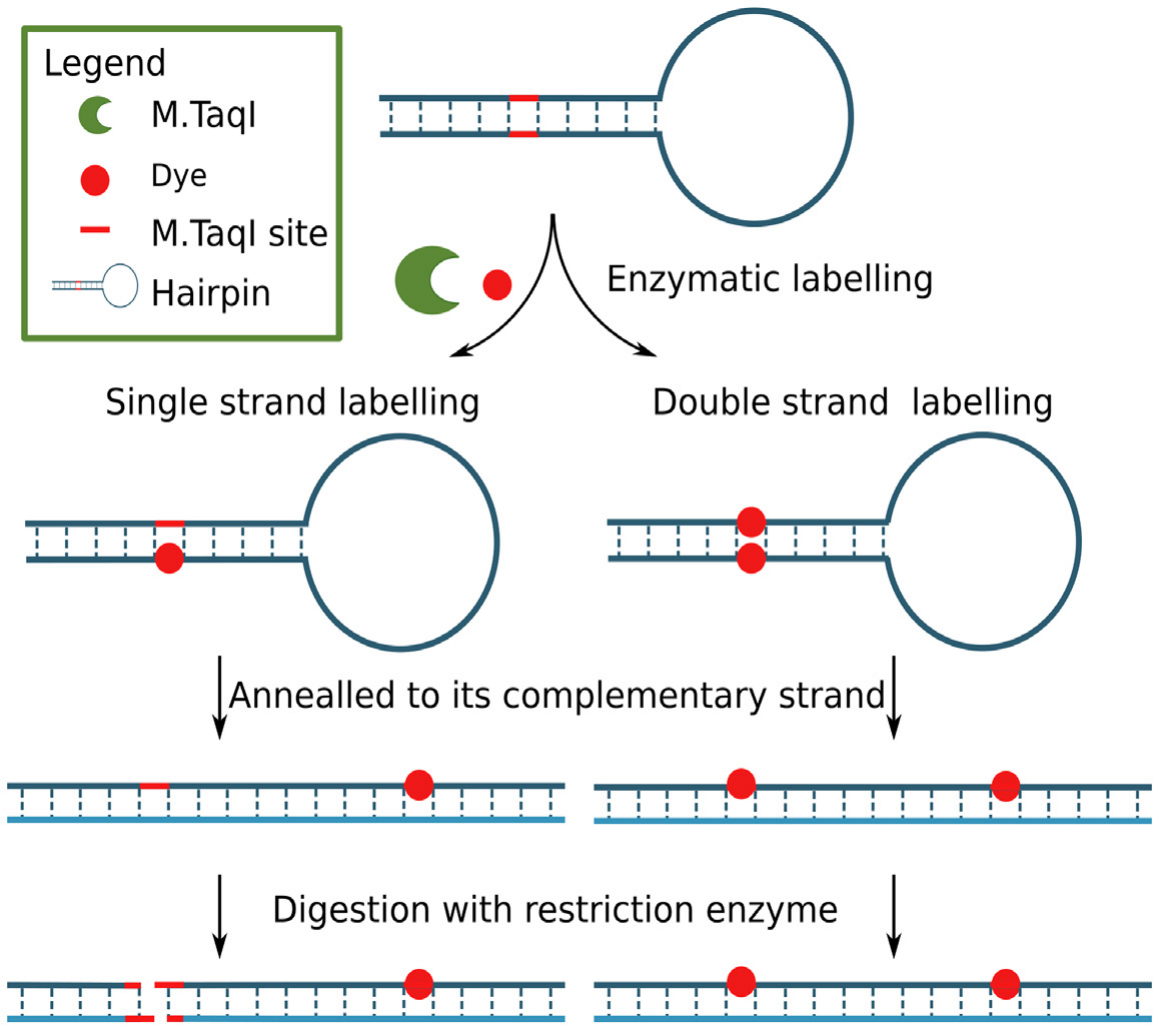
Schematic illustration of hairpin experiment designed for single strand or double strand labeling testing. A 96 base long hairpin containing only one M.TaqI recognition site (TCGA) was designed. After enzymatic labeling, the hairpin was annealed to its complementary strand, after which, the double strand DNA molecule was digested with R.Taq^α^I (NEB). In the case of single strand labeling, the double strand molecule would be digested at one site; in the case of double strand labeling, the double strand molecule would remain intact.

In order to address this experimentally, we implemented two experiments using a 96 bp DNA hairpin, Figure 6. The hairpin was subjected to our one-pot labeling reaction, using the Ado-6-amine cofactor and Atto647N NHS ester. Following labeling, a complementary strand was annealed to the hairpin, the newly formed DNA duplexes were challenged with a restriction enzyme. Since hemi-alkylation at a site prevents the endonuclease activity of R.TaqI, we are subsequently able to assess the extent of modification that occurred on the original hairpin.

The restriction assay (Supplementary Figure S12) implies that a mixed population of one- and two-strand labeling (or alkylation) occurs in the enzymatic reaction. To further confirm this result and to better understand the type of modification bound to the DNA, we analyzed the labeled hairpins using mass spectrometry. The result is conclusive (Supplementary Figure S13), showing that we see a single fluorophore per labeling site and, where both palindromes of the M.TaqI recognition sequence are modified, one carries a fluorophore, the other is alkylated but has no fluorophore attached. Hence, in our counter assay, we expect plasmids that are fully-labeled to carry a maximum of four fluorophores. Greater numbers of fluorophores associated with the plasmids likely indicate off-site labeling by the M.TaqI or non-specific binding of the fluorophore to the DNA. This is consistent with our observations of fluorophore numbers and specificity on the combed PCR fragments in Figure 5.

The DNA methyltransferase enzymes have an important role to play as tools for fluorescent labeling, DNA capture and sorting and broader DNA functionalization. This is a direct result of their unique sequence-specific, non-damaging mechanism that allows quick and easy DNA modification at pre-determined sites. Here, we have developed an approach that allows the functionalization of DNA using a range of DNA methyltransferase enzymes, a broad range of functionalities and a simple one-pot reaction. We have shown that DNA labeling is specific, efficient and that a single modification is to be expected at a given target site for the enzyme. We see consistently high labeling efficiencies on both linear DNA molecules (such as the 96 bp synthetic oligonucleotide and the 5 kb PCR fragment we studied) and on plasmid DNA molecules, such as those we have used in our single-moleucle imaging assays. Off-site labeling is undetectable in the hairpin DNA oligonucleotides and occurs with a frequency of ∼0.4 labels/kb on longer DNA. Our one-pot labeling approach gave a significant improvement in fluorophore conjugation efficiency compared with previous approaches and can be readily extended to screen the methyltransferase-directed transfer of a multitude of functional groups directly to DNA from a single AdoMet analogue (Ado-6-amine).

## Supplementary Material

Supplementary Data
