# Supplementary material for: A general strategy for direct, enzyme-catalyzed conjugation of functional compounds to DNA
Source: Nucleic Acids Res. 2018 Mar 13;46(11):e64. doi: 10.1093/nar/gky184 (PMC6009647; doi:10.1093/nar/gky184)
Supplement: Supplementary Data [file gky184_supplemental_figures.pdf]

A general strategy for direct, enzyme-catalyzed conjugation of functional compounds to DNA

Jochem Deen, Su Wang, Sven Van Snick, Volker Leen, Kris Janssen, Johan Hofkens, and Robert K. Neely

Supplementary information

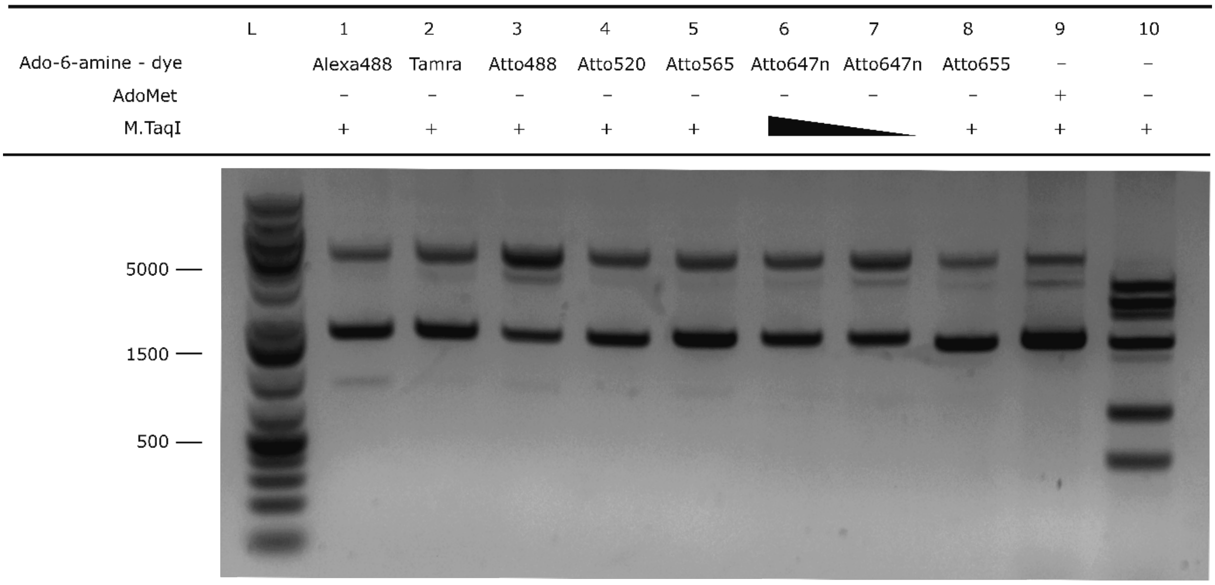

Figure S1: Restriction assay on amine cofactor coupled to one of several NHS-ester dyes. Final concentration of 0.21mg/ml (or 0.14mg/ml for lane 7) of M.TaqI and 200µM of ado-6-amine-dye were used in the reaction.

	L	1	2	3	4	5	6	7	8	9
Ado-6-amine - dye		Alexa488	Tamra	Atto488	Atto520	Atto565	Atto647n	Atto655	–	–
AdoMet		–	–	–	–	–	–	–	+	–
M.TaqI		+	+	+	+	+	+	+	+	+

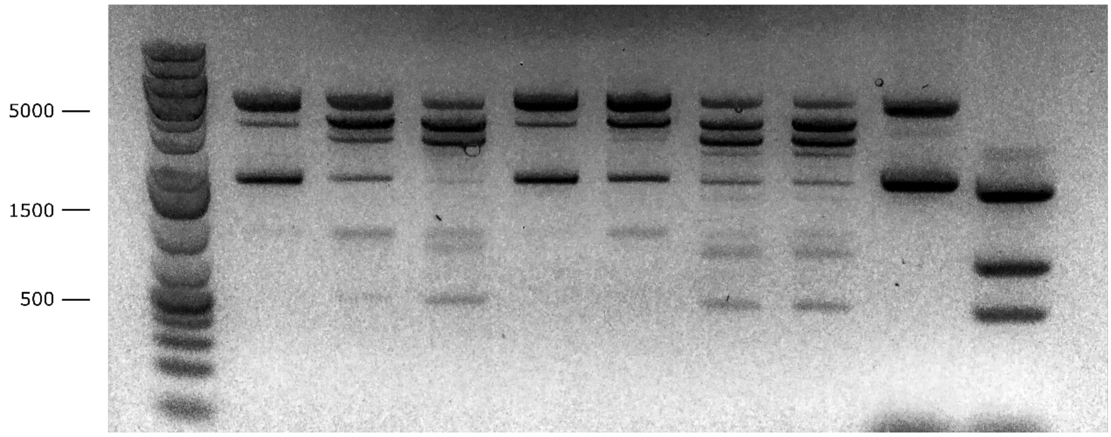

Figure S2: Restriction assay on amine cofactor coupled to one of several NHS-ester dyes. Final concentration of 0.175mg/ml of M.TaqI and 200µM of ado-6-amine-dye were used in the reaction. The lower concentration of M.TaqI used here resulted in only partial protection of the DNA against restriction using some of the dyes.

	L	1	2	3	4	5	6	7	8	9	10	11	12
cofactor		Biotin-NHS	BiotinNHS	Biotin-NHS	PEG	PEG	PEG	–	–	–	–	–	–
AdoMet		–	–	–	–	–	–	–	–	+	+	–	–
M.TaqI		+	+	+	+	+	+	+	+	–	–	–	–
R.Taq$^{\alpha}$I		+	+	+	+	+	+	–	+	+	–	–	+
Incubation time (min)		0	30	120	0	30	120	/	/	/	/	/	/

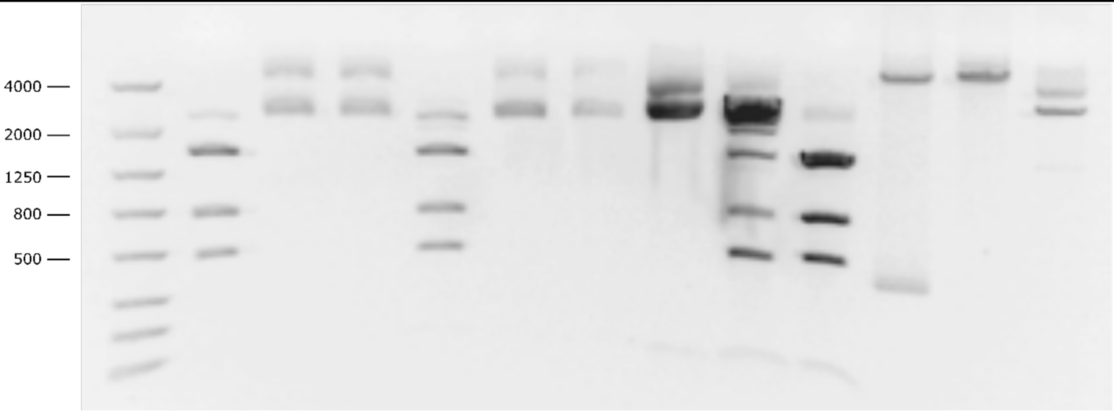

Figure S3: Restriction assay on amine cofactor coupled to biotin or polyethylene glycol (4000MW). A final concentration of 0.175mg/ml of M.TaqI and 200µM of ado-6-amine was used in the reaction.

	L	1	2	3	4	5	6	7	8	9	L
Ado-6-amine - Atto647N		+	+	+	+	+	Ado-6-amine	–	–	–	
AdoMet		–	–	–	–	–	–	+	+	–	
M.TaqI							+	+	–	+	

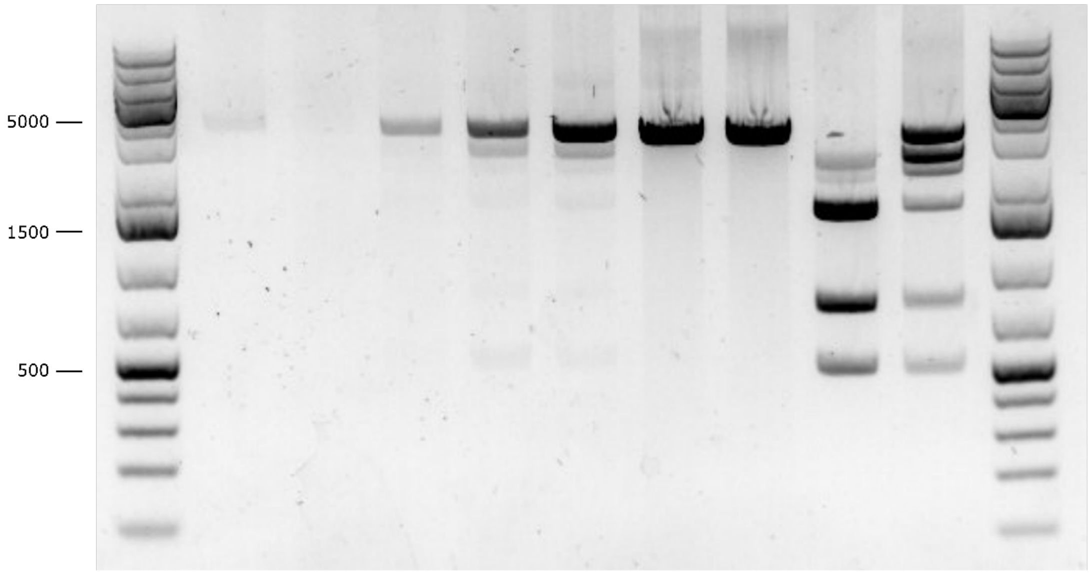

Figure S4: Restriction assay on amine cofactor coupled to Atto647N NHS-ester dye with various M.TaqI methyltransferase concentrations. pUC19 plasmid DNA was linearized by SalI prior to the restriction assay. From lane 1 till lane 5, a 2x dilution series of M.TaqI (final concentration starting from 0.38mg/ml) was performed.

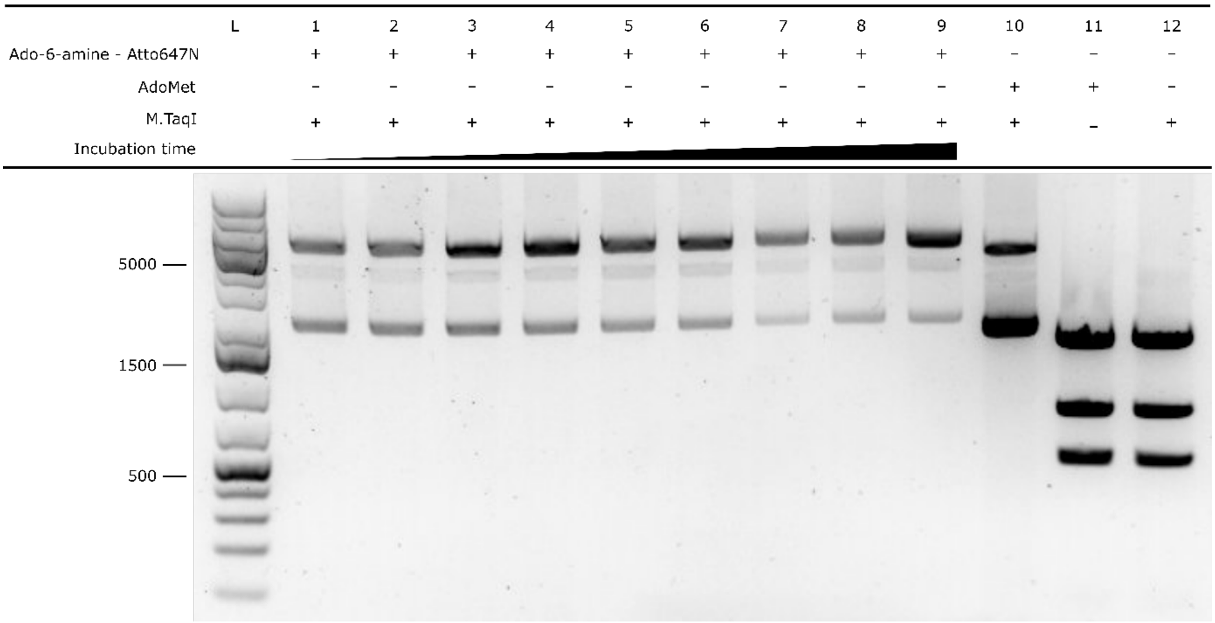

Figure S5: Restriction assay on amine cofactor coupled to Atto647N NHS-ester dye with various incubation time. All the samples were incubated at 60 ºC. From lane 1 to lane 9, the incubation times were: 1’, 2’, 5’, 10’, 15’, 20’, 30’, 60’, and 120’ respectively. All the samples showed complete labelling. Final concentration of M.TaqI was 0.24mg/ml.

	L	1	2	3	4	5	6	7	8	9	10	11	12
Ado-6-amine - Atto647N		+	+	+	+	+	+	-	-	-	-	-	-
AdoMet		-	-	-	-	-	-	+	+	+	+	-	-
M.FokI								+	+	-	-	+	+
R.FokI		+	+	+	+	+	+	-	+	-	+	-	+

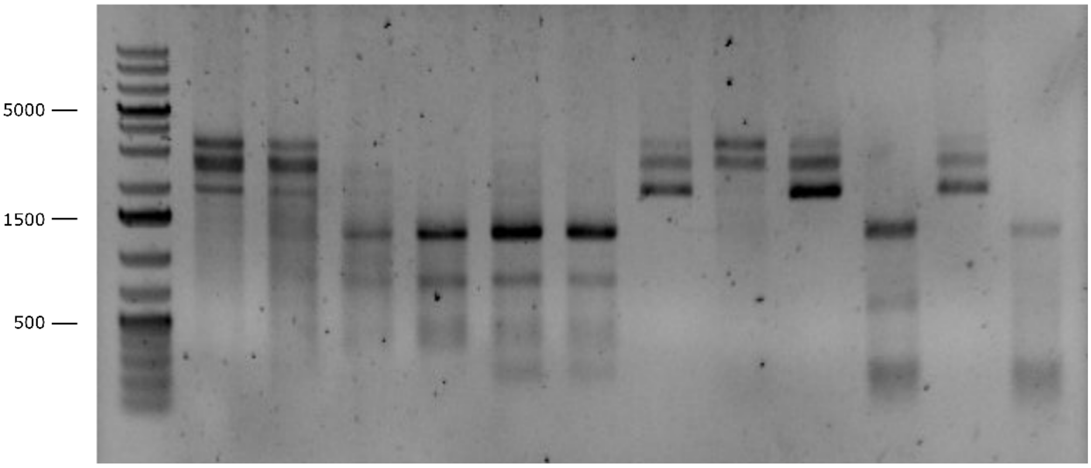

Figure S6: Restriction assay on amine cofactor coupled to Atto647N NHS-ester dye with various M.FokI methyltransferase concentrations. Lanes 1 to 6 show a 2x dilution series of M.FokI (starting concentration from 2.5mg/ml)

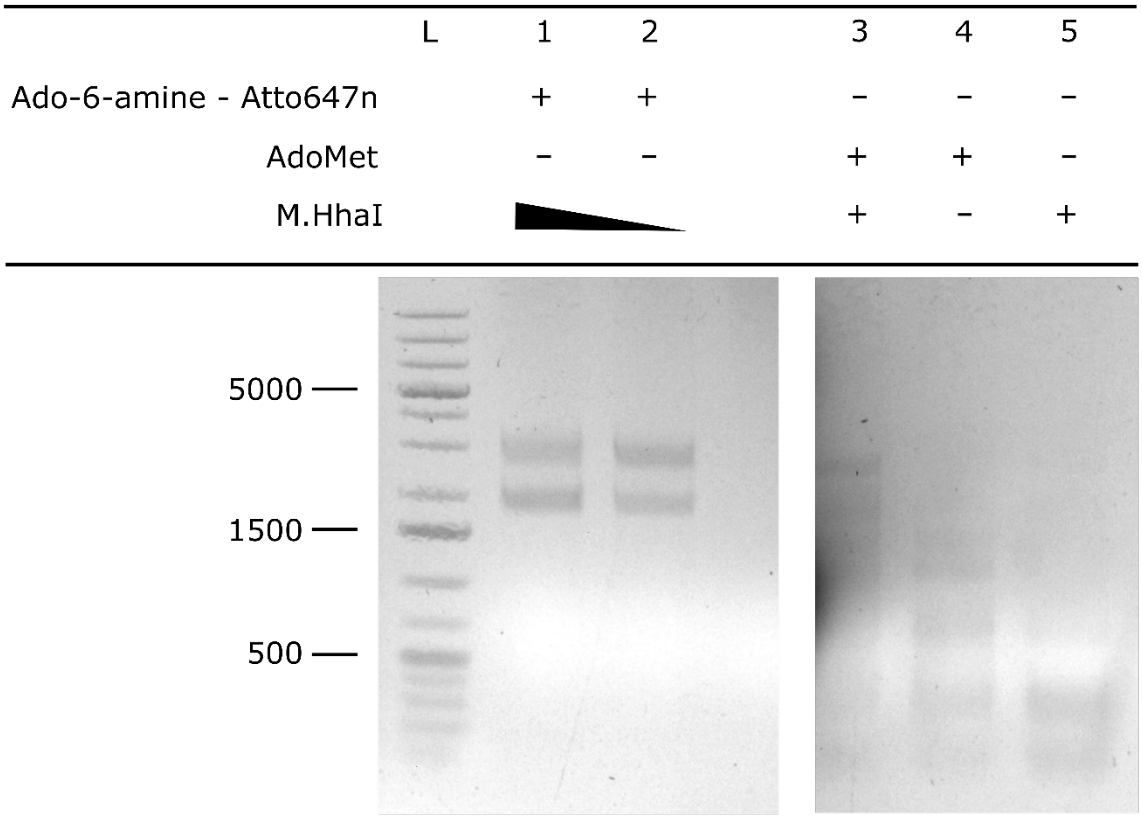

Figure S7: Restriction assay on amine cofactor coupled to Atto647N NHS-ester dye with M.HhaI methyltransferase enzyme. The reaction mixture was incubated at 37 ºC, and restricted with R.HhaI. M.HhaI enzyme concentration decreased from lane 1 (final enzyme concentration of 1mg/ml) to lane 2 (final enzyme concentration of 0.78mg/ml). The middle part of the gel was cut out because those were samples from another experiment.

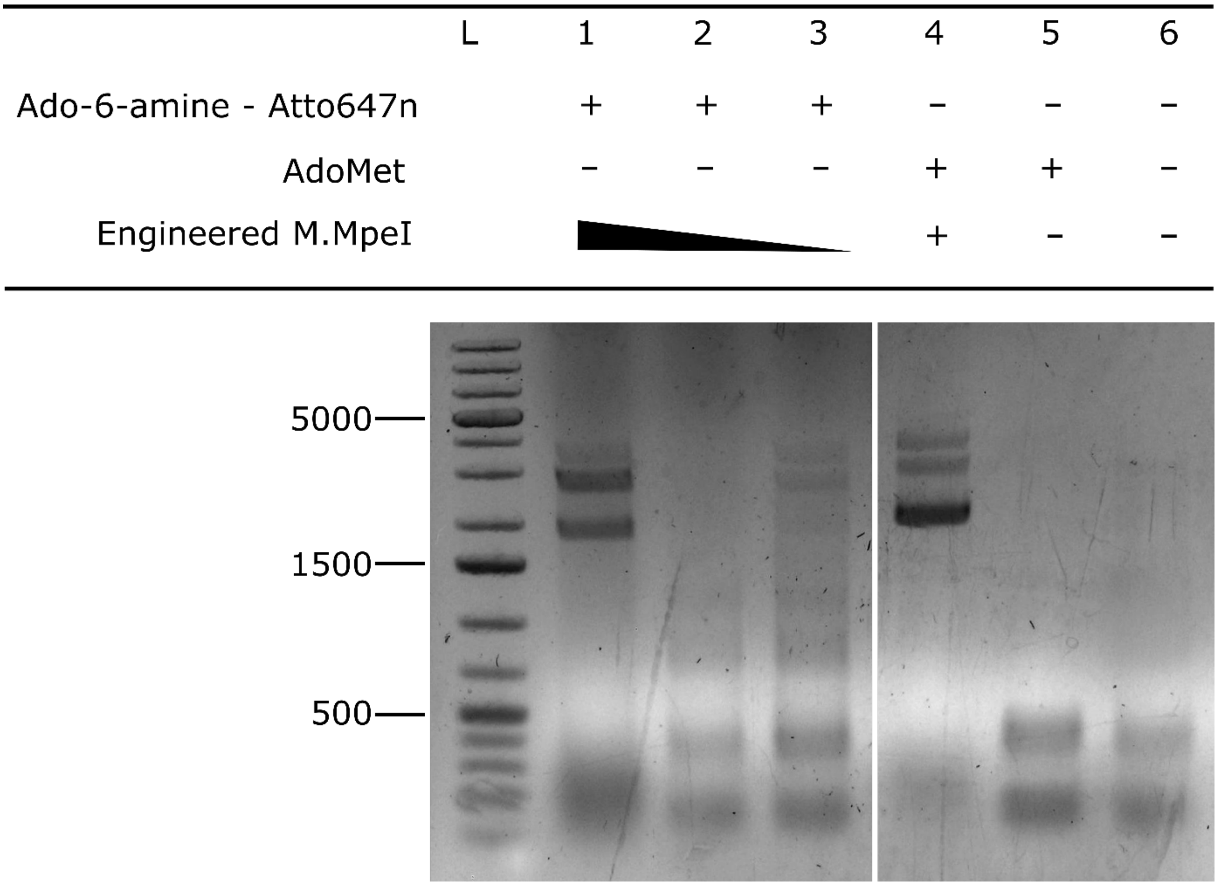

Figure S8: Restriction assay on amine cofactor coupled to Atto647N NHS-ester dye with M.MpeI methyltransferase enzyme. The reaction mixture was incubated at 37 ºC, and restricted with R.HhaI. M.MpeI enzyme concentration decreased from lane 1 to lane 3 (0.13mg/ml, 0.09mg/ml, 0.04mg/ml respectively). The middle part of the gel was cut out because those were samples from another experiment.

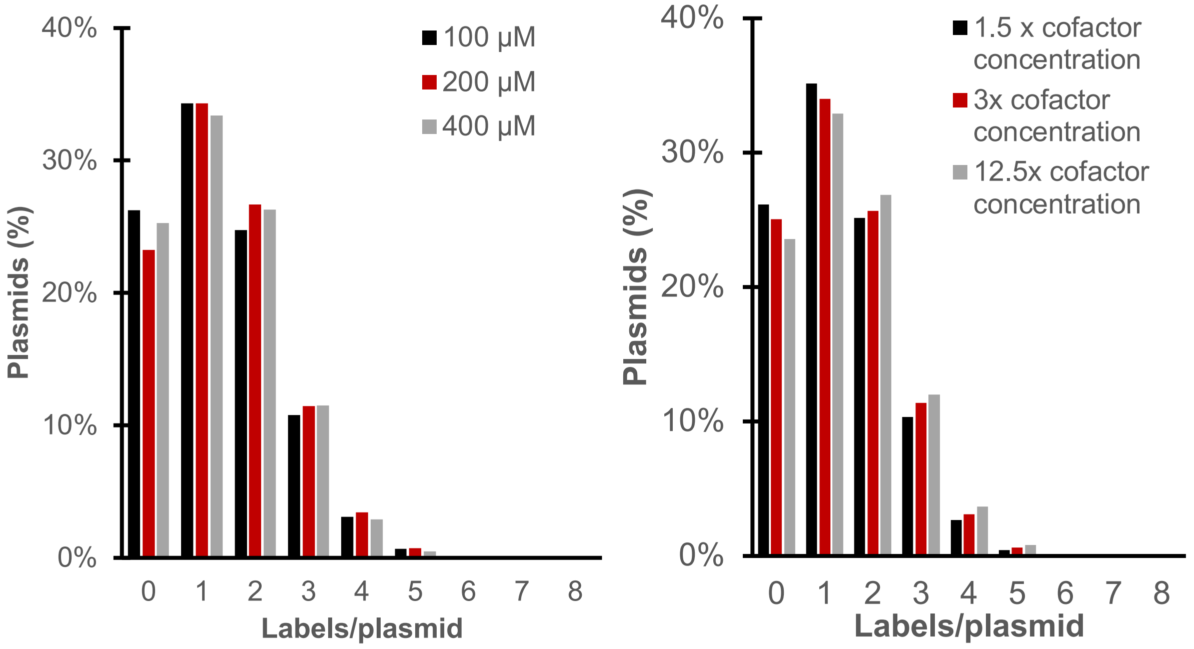

Figure S9: Histogram of number of localized Atto647N labels per pUC19 plasmid for different cofactor concentrations (left) and different dye concentrations (right).

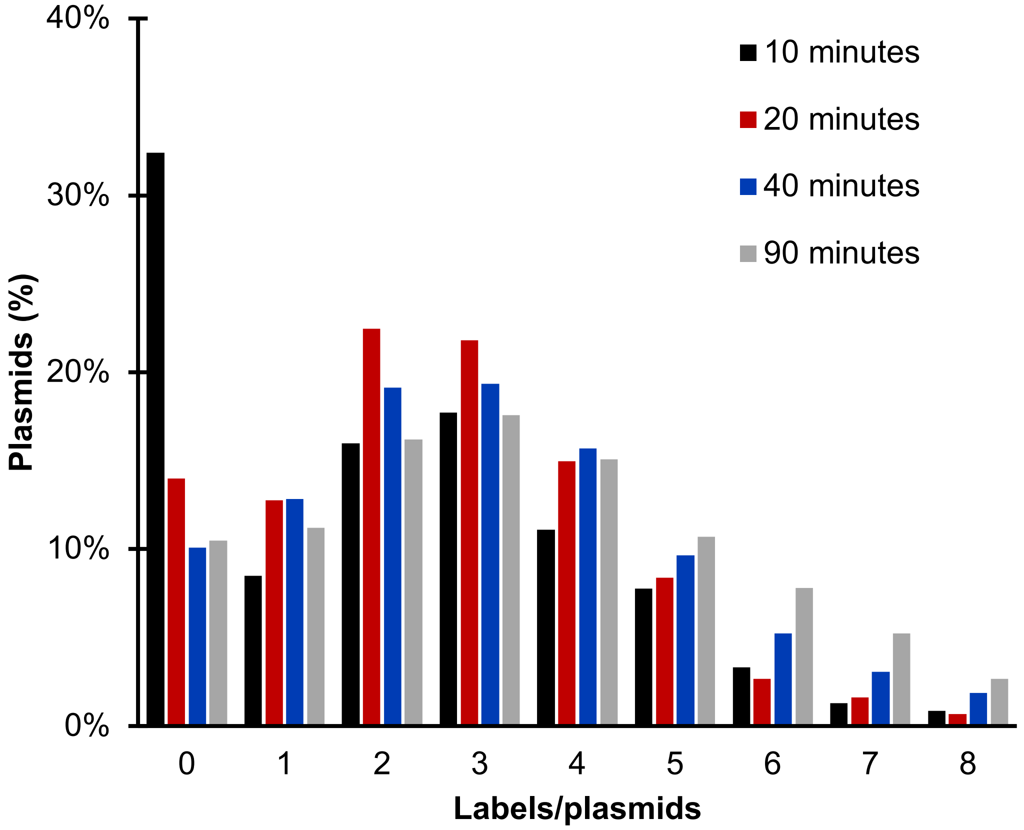

Figure S10: Histogram of number of localized Atto647N labels per pUC19 plasmid with various incubation time between AdoMet analogue and Atto647N NHS ester.

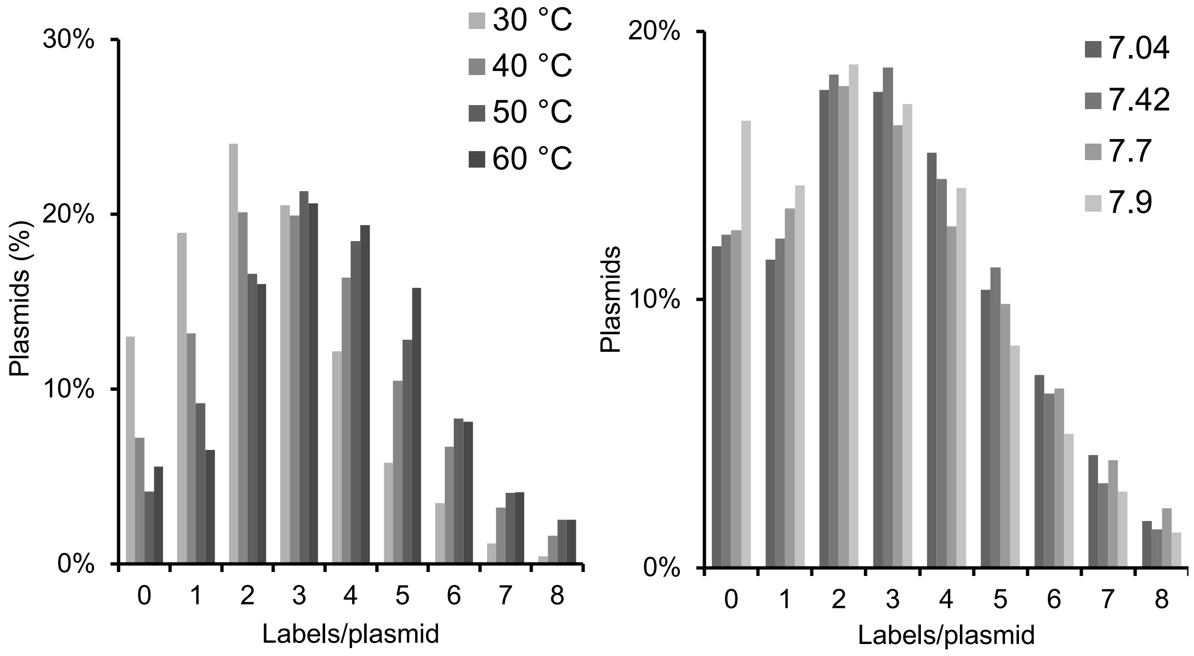

Figure S11: Histogram of number of localized Atto647N labels per pUC19 plasmid at different temperature (left) and different pH (right).

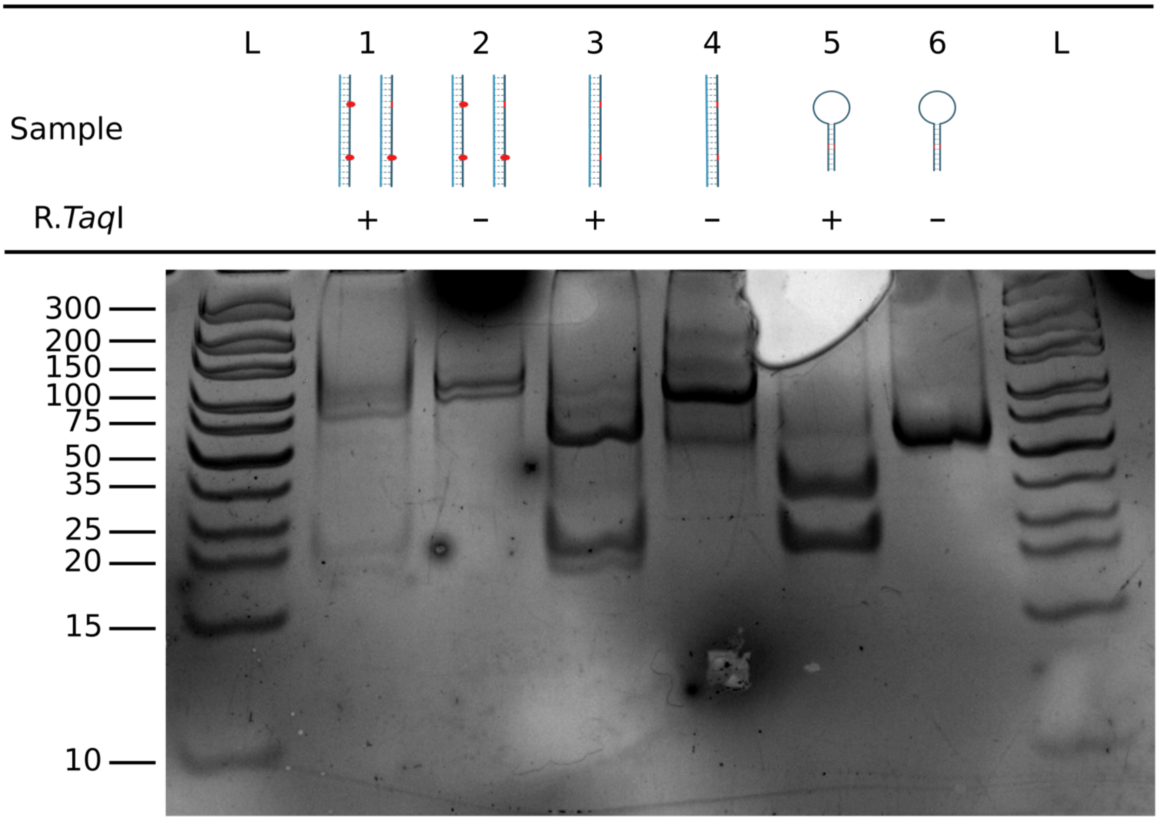

Figure S12: Restriction gel of hairpin experiment. The samples (3µM) were labeled with M.TaqI enzyme with a final concentration of 0.24mg/ml and ado-6-amine-dye concentration of 0.38µM. Lane 1 contains three bands: ~100bp, ~80bp and ~20bp. They represent undigested DNA molecule (double strand labeled hairpin), the big part and the small part of one-cut DNA molecule (single strand labeled hairpin). Lane 2 is the same sample without restriction. Lane 3 till lane 6 are controls. The lowest band in lane 4 shows the hairpin instead of double strand molecule.

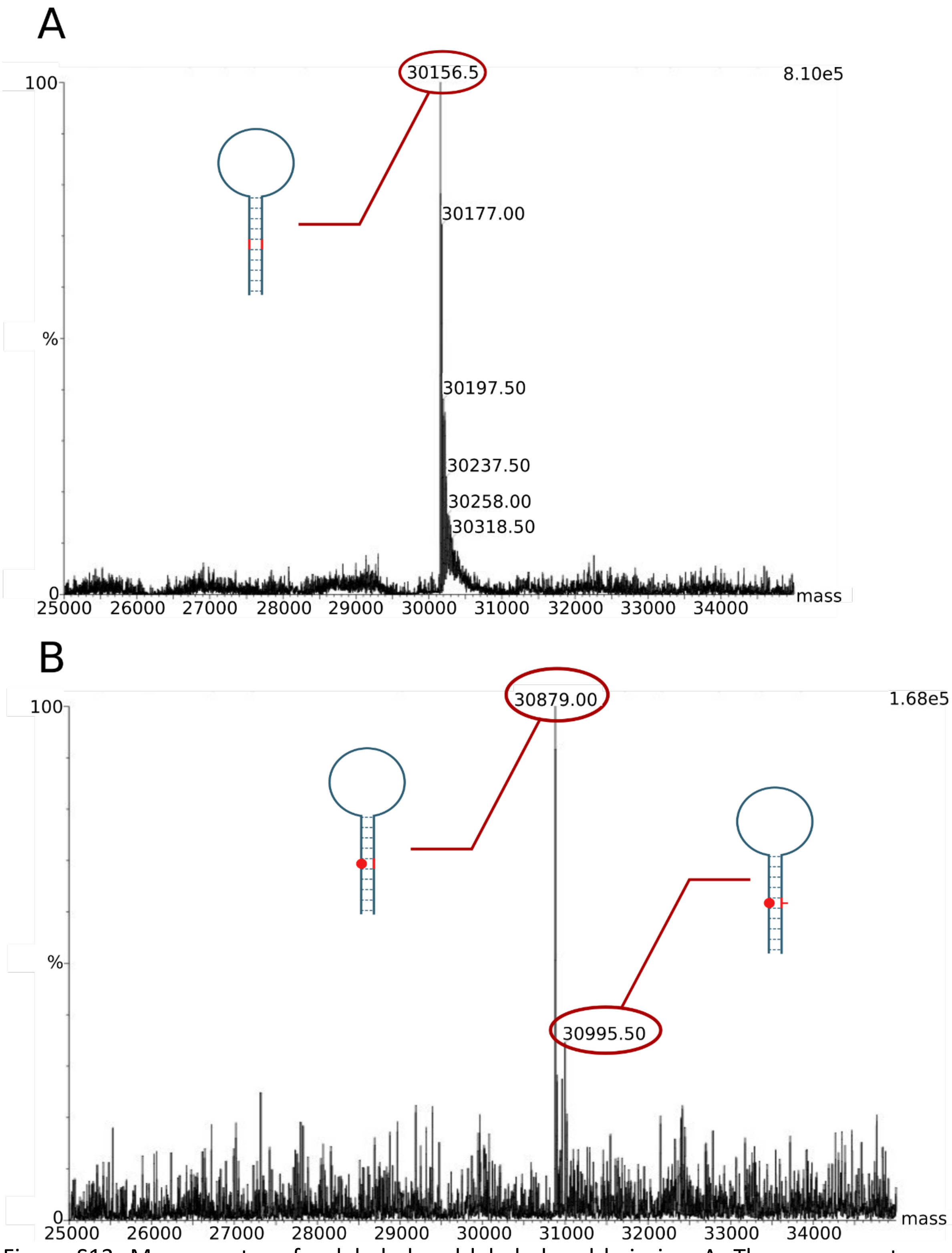

Figure S13: Mass spectra of unlabeled and labeled and hairpins. A: The mass spectrum of unlabeled hairpin. B: The mass spectrum of labeled hairpin. The unlabelled hairpin has a mass of 30156 Da, the labelled hairpin has two populations with mass of 30879 Da and 30995.5 Da respectively. The major peak in B corresponds to the hairpin plus Atto647N (628.9 Da) and ado-6-amine linker (96.2 Da), 30880.6 Da. The mass difference between the minor peak and major peak in figure B is 116.6, this corresponds to the mass of the addition of the ado-6-amine sidechain plus a sodium ion (which is present in the buffer).
